# Practitioner's Bookshelf

**Published:** 1892-05-14

**Authors:** 


### PRACTITIONERS' BOOKSHELF.

### CATECHISM SERIES—PUBLIC HEALTH.*

Part III.—Sewage and its Treatment

Are dealt with at some length. Among errors of omission, commission, and incompatible statements may be instanced the following: The definition of "drain" is not that of the Public Health Act, 1875. It is stated that the velocity of house drains should be 3 to 10 feet per second, but that a flow faster than 4 feet is apt to cut the pipes; later on it is said the velocity in house drains should be 4½ feet per second. The fall necessary to give a velocity of 3 feet per second in a 4-in. pipe is said to be 1 in 92—Louis Parkes gives 1 in 40 as necessary for this velocity, and Edmund Parkes says a 4-in. drain should have a fall of 1 in 30. Apparently the only form of water-closet recognised is the valve closet. No mention is made of a disconnecting chamber. The statement that the excess of death-rate in Brighton and Liverpool, over 17·0 per 1,000 is due to the forcing of street gullies and house traps by sewer gas while the outfall sewers are tide-locked—all other influences being ignored—seems hardly justifiable, and is an example of jumping at conclusions.

Part IV.—Vital Statistics.

The paragraphs on estimation of population in the intercensal period, and the calculation of a death-rate, are full of errors. No mention is made of the logarithmic mode of estimating population. As to the death-rate example given, that of Edinburgh in 1883—having arrived at the conclusion, in a loose way, that the rate for the year 1883 is 18.6, it is stated "to this, however, a little should be added to make up for the extra quarter—from April to June inclusive—say 19.0 per 1,000." This is absolutely incorrect and misleading, and encourages those loose statistical methods which should be strictly avoided. Again, what is a student (or anyone else) to make of the following?—"What is a birth-rate?" "It is the total births per year, divided into the rate per 1,000 of the population living in the district at all ages." The substitution of erysipelas for diarrhœa among the seven zymotic diseases is novel. If the other parts of the series are on a par with these two the hope expressed in the preface "that the method of question and answer here adopted will aid the student in preparing his work for examinations," and that "the book will be a great and useful means of self-tuition," is hardly likely to be realised.

* "Catechism Series. Public Health. Part III., Sewage and its Treatment; and Part IV., Vital Statistics." (E. and S. Livingstone, Edinburgh.)

### THE PULSE.

This little monograph* very fairly embodies the current views of clinicians and physiologists on the pulse. While the subject is treated elementarily, and the matter is in parts almost redundant, it contains a great deal of information that will help the practitioner. The introduction and perfection of the various methods of physical investigation have tended to lessen the importance of the pulse as a diagnostic sign in the eyes of students, but in practice a complete knowledge of what the pulse implies is more immediately useful than almost any other physical sign. Dr. Ewart's work will therefore be welcomed by many practitioners, as well as students, as a valuable aid in their profession.

* "How to feel the Pulse, and What to feel in it." By Wm. Ewart, M.D., F.R.C.P. Illustrated, pp. 112. (London: Baillière, Tindall, and Cox.)

